# In Situ Synthesis of a Tumor-Microenvironment-Responsive Chemotherapy Drug

**DOI:** 10.3390/pharmaceutics15041316

**Published:** 2023-04-21

**Authors:** Xiupeng Wang, Ayako Oyane, Tomoya Inose, Maki Nakamura

**Affiliations:** 1 Health and Medical Research Institute, National Institute of Advanced Industrial Science and Technology (AIST), Central 6, 1-1-1 Higashi, Tsukuba 305-8566, Ibaraki, Japan; 2 Nanomaterials Research Institute, National Institute of Advanced Industrial Science and Technology (AIST), Central 5, 1-1-1 Higashi, Tsukuba 305-8565, Ibaraki, Japan

**Keywords:** mesoporous silica (MS) doped with Cu (MS-Cu), disulfiram (DSF), chemotherapy, cancer, in situ synthesis

## Abstract

Current chemotherapy still suffers from unsatisfactory therapeutic efficacy, multi-drug resistance, and severe adverse effects, thus necessitating the development of techniques to confine chemotherapy drugs in the tumor microenvironment. Herein, we fabricated nanospheres of mesoporous silica (MS) doped with Cu (MS-Cu) and polyethylene glycol (PEG)-coated MS-Cu (PEG-MS-Cu) as exogenous copper supply systems to tumors. The synthesized MS-Cu nanospheres showed diameters of 30–150 nm with Cu/Si molar ratios of 0.041–0.069. Only disulfiram (DSF) and only MS-Cu nanospheres showed little cytotoxicity in vitro, whereas the combination of DSF and MS-Cu nanospheres showed significant cytotoxicity against MOC1 and MOC2 cells at concentrations of 0.2–1 μg/mL. Oral DSF administration in combination with MS-Cu nanospheres intratumoral or PEG-MS-Cu nanospheres intravenous administration showed significant antitumor efficacy against MOC2 cells in vivo. In contrast to traditional drug delivery systems, we herein propose a system for the in situ synthesis of chemotherapy drugs by converting nontoxic substances into antitumor chemotherapy drugs in a specific tumor microenvironment.

## 1. Introduction

With approximately 20 million new cancer cases and approximately 10 million cancer deaths every year, cancer ranks as a leading cause of death worldwide [1]. Surgery, chemotherapy, radiotherapy, and immunotherapy are the most common treatments for cancer. Chemotherapy uses drugs to destroy rapidly growing and dividing cancer cells throughout the body; thus, it is still one of the best ways to treat various cancers. However, the systemic administration of chemotherapy drugs is always accompanied by low treatment efficacy and system toxicity due to off-target effects. Therefore, it is desirable that chemotherapy drugs should be delivered and confined to the tumor microenvironment. Despite significant progress, drug delivery systems for targeted chemotherapy still face many challenges, including unsatisfactory therapeutic efficacy and off-target toxicity [2,3,4,5,6,7,8,9]. To meet this challenge, one promising strategy is the synthesis of chemotherapy drugs in situ by converting nontoxic substances into antitumor chemotherapy drugs in a specific tumor microenvironment.

Although considerable progress in early diagnosis and new therapies have markedly improved the survival rate of cancer patients, the five-year survival rate for stage IV patients is still very low. For instance, the five-year survival rates for stage IV breast cancer, rectal cancer, and colon cancer patients are only 28%, 15%, and 11%, respectively [10]. Therefore, it is highly desirable to develop more effective therapies for cancer. However, the development of new therapies for cancer is extremely challenging because of the low success rate, high cost, high risk, intense competition, and long research and clinical test periods.

Repurposing approved medicines for cancer treatment is a more effective strategy than introducing new medicines [11], owing to its higher approval rate, shorter development timeline, lower development cost, and the more comprehensive information available, including formulation, dose, safety, tolerability, and pharmacology. Recently, some approved medicines including DSF, metformin, and aspirin have shown antitumor efficacy [12,13,14]. In particular, DSF, a drug for alcoholism treatment approved by the FDA over 70 years ago, has shown Cu^2+^-dependent antitumor efficacy [15]. The -S-S- bonding in DSF can be oxidized and chelated by Cu^2+^ to form bis(diethyldithiocarbamate)-copper complexes (CuETs), which show a broad-spectrum antitumor efficacy against a variety of tumors by disrupting essential signaling pathways. CuETs also show synergistic antitumor efficacy with traditional chemotherapy drugs, including cisplatin, gemcitabine, doxorubicin, and 5-fluorouracil [16,17,18,19,20]. CuETs induce nuclear protein localization-4 (NPL4) aggregation after their binding, prevent the p97-NPL4-ubiquitin fusion degradation protein 1 pathway, induce a complex cellular phenotype, and cause cell death [15].

However, the clinical application of DSF in cancer treatment is considerably hampered by inadequate Cu^2+^ in the tumor microenvironment. Although Cu is an essential trace element for many organisms, extra external Cu triggers cell death by causing mitochondrial protein aggregation, and its nonspecific biodistribution in the body may cause unintended side effects and toxicity [21,22]. Orally administered Cu^2+^-containing compounds that are commonly used for treating Cu^2+^ deficiency in clinical practice are liable to accumulate in normal tissues and thus may cause serious side effects and toxicity [23,24]. Therefore, to realize the full therapeutic potential of DSF-based chemotherapy in cancer treatment, it is essential to increase the local Cu^2+^ concentration in the tumor microenvironment with minimal Cu^2+^ accumulation in normal tissues.

For this purpose, PEG-Cu-DSF nanocomplexes were developed to deliver both DSF and Cu^2+^ by a same nanoparticle into tumors. After the intravenous or intratumoral administration of PEG-Cu-DSF nanocomplexes, DSF and Cu^2+^ were rapidly released and transformed into cytotoxic CuETs in the endogenous weakly acidic tumor microenvironment, thus showing high chemotherapeutic efficacy [22,25]. However, DSF is approved only for oral administration. Therefore, it is desirable to use an exogenous copper supply system to tumors together with the FDA-approved oral administration of DSF.

Biocompatible MS nanospheres are good carriers of metal ions. Metal ions can break Si–O–Si linkages in MS and coordinate with the resulting non-bridging oxygens; therefore, metal ion doping increases the dissolution and degradation rates of MS [26,27,28,29,30,31]. Various metal ions, including Ca^2+^, Mg^2+^, Zn^2+^, Mn^x+^, Fe^x+^, Sr^2+^, Cu^2+^, Al^3+^, Ti^4+^, Zr^4+^, Ag^+^, etc. have been doped in MS to achieve specific functions [26,27,28,29,30,31]. The metal ions released from MS during degradation play a valuable role in regulating osteo/odontogenesis, angiogenesis, antibacterial properties, the tumor microenvironment, and the immune system [26,27,28,29,30,31,32,33].

In this study, we fabricated MS-Cu nanospheres and PEG-MS-Cu nanospheres as exogenous copper supply systems to tumors. Together with oral DSF administration, intratumoral MS-Cu nanospheres administration and intravenous PEG-MS-Cu nanospheres administration significantly inhibited tumor growth in vivo. Therefore, orally administered DSF converted into antitumor chemotherapy drugs (CuETs) in vivo with the aid of the present exogenous copper supply system; thus, this is a promising strategy for cancer chemotherapy.

## 2. Materials and Methods

### 2.1. Synthesis of MS-Cu Nanospheres

MS-Cu-1 nanospheres were synthesized by adding tetraethoxysilane (TEOS, FUJIFILM Wako Pure Chemical Corporation, Minato City, Japan) dropwise into a cetyltrimethylammonium p-toluenesulfonate (CTAT, Sigma-Aldrich, St. Louis, MO, USA) aqueous solution supplemented with triethanolamine (TEA, Sigma-Aldrich, St. Louis, MO, USA) under vigorous stirring at 75 °C. After adding TEOS, copper nitrate trihydrate (FUJIFILM Wako Pure Chemical Corporation, 0.16 g/mL, Minato City, Japan) was added dropwise with vigorous stirring. The quantities of TEOS, TEA, CTAT, Cu(NO_3_)_2_·3H_2_O, and water were 1.5 mL, 1 g, 0.4 g, 0.16 g, and 20 mL, respectively. After 4.5 h, the precipitate was collected after centrifugation, washed with ultrapure water and ethanol, dried at 75 °C, and calcined at 550 °C for 5 h. MS-Cu-2 and MS-Cu-3 nanospheres were synthesized by the same method as in the synthesis of MS-Cu-1 nanospheres, except that 0.6 g and 0.4 g of TEA were added, respectively.

### 2.2. Synthesis of PEG-MS-Cu Nanospheres

First, 500 mg of MS-Cu-1 nanospheres were dispersed in 40 mL of ethanol. Then, 5 mL of 3-aminopropyl triethoxysilane (FUJIFILM Wako Pure Chemical Corporation, Minato City, Japan) was added and stirred at 25 °C for 1 d in the dark. The products were collected by centrifugation, then washed with ethanol twice. The collected nanospheres were dispersed in 40 mL of 2-(N-morpholino)ethanesulfonic acid monohydrate solution (0.1 mol/L, pH = 5.5, FUJIFILM Wako Pure Chemical Corporation, Minato City, Japan). Then, 65.92 mg of 1-[3-(dimethylamino)propyl]-3-ethylcarbodiimide hydrochloride (Sigma Aldrich, St. Louis, MO, USA), 36.83 mg of N-hydroxysuccinamide (Sigma Aldrich, St. Louis, MO, USA), and 146.4 mg of PEG acid disulfide (Polypure, MW = 915.1) were added slowly and stirred at room temperature for 12 h. The products were collected by centrifugation, washed with ultrapure water twice, and freeze-dried to prepare the PEG-MS-Cu nanospheres.

### 2.3. Characterization of MS-Cu Nanospheres

The MS-Cu nanospheres were characterized using a transmission electron microscope (TEM, JEOL, Akishima, Japan) and a powder X-ray diffractometer with CuKα X-rays (RINT 2500, Rigaku, Tokyo, Japan). The nitrogen gas (N_2_) adsorption–desorption isotherm of the MS-Cu nanospheres was measured using a surface area and porosity analyzer (TriStar II, Micromeritics). The BET specific surface areas and pore size distributions were calculated. The Cu/Si molar ratios of the MS-Cu nanospheres were examined by dissolving the nanospheres in 1M NaOH and 2M HCl, followed by inductively coupled plasma–atomic emission spectrometry (ICP-AES, Hitachi High-Technologies, Tokyo, Japan). In vitro copper ion release was studied by immersing nanospheres (1 mg/mL) in an acetate buffer (pH = 5) at room temperature. At certain time intervals, the supernatants were collected, and new buffers were supplemented. The copper ion release was analyzed by ICP-AES. The stability of PEG-MS-Cu nanospheres was tested by performing dynamic light scattering analysis (Otsuka Electronics, Osaka, Japan).

### 2.4. In Vitro Cytotoxicity of MS-Cu Nanospheres and DSF; In Vitro Reactive Oxygen Species (ROS) Generation

Mouse oral squamous cell carcinoma 1 (MOC1) and MOC2 cells (Kerafast, Boston, MA, USA) were seeded onto 96-well plates at 1 × 10^4^ cells/0.1 mL/well and cultured for 24 h. Then, only MS-Cu nanospheres, only DSF, and a combination of MS-Cu nanospheres and DSF were added to the medium at various concentrations up to 1.0 μg/mL, and the cells were cultured for 24 h. The number of cells was assayed using a CCK-8 kit (Dojindo Molecular Technologies, Rockville, MD, USA) according to the manufacturer’s instructions. The ROS generation was analyzed using a DCFDA/H2DCFDA-Cellular ROS Assay Kit (Abcam, Cambridge, UK). MOC2 cells (2.5 × 10^5^ cells/mL) were seeded in 96-well plates, cultured overnight, and incubated with nanospheres for 6 h. After incubating the cells with DCFDA (30 μmol) for 45 min, the fluorescence of DCF (Ex/Em = 492 nm/530 nm) was measured using the microplate reader.

### 2.5. In Vitro Safety of MS-Cu and PEG-MS-Cu

The in vitro safety of MS-Cu and PEG-MS-Cu was tested using fibroblastic NIH3T3 cells (NIH3T3-3-4, Riken Bio Resource Center, Kyoto, Japan). A total of 1 × 10^4^ cells/mL cells were seeded in 96-well plates and cultured overnight. After incubating the cells with nanospheres for 24 h, the viability of the cells was tested using a CCK-8 kit.

### 2.6. In Vivo Antitumor Efficacy of Combined Oral Administration of DSF and Intratumoral Administration of MS-Cu Nanospheres

First, 5 × 10^5^ MOC2 cells in 0.05 mL of phosphate buffered saline (PBS) were injected into the left hind legs of female C57BL/6J mice (CLEA Inc., Tokyo, Japan, 6 weeks old). Then, the mice were orally administered daily with DSF (1.5 mg/mouse) from d3 to d9 in combination with intratumoral administration of MS-Cu nanospheres (2 mg/mouse) on d4 and d6. The mice administered with only DSF and without any treatment were used as controls. Tumor size was measured using a digital caliper. Tumor volume was calculated as ½ × (longest dimension) × (perpendicular dimension)^2^.

### 2.7. In Vivo Antitumor Efficacy of Combined Oral Administration of DSF and Intravenous Administration of PEG-MS-Cu Nanospheres

First, 1 × 10^6^ MOC2 cells in 0.1 mL of PBS were injected into the left hind legs of mice. Then, the mice were orally administered daily with DSF (1.5 mg/mouse) from d3 to d11 in combination with intravenous administration of PEG-MS-Cu nanospheres (1.5 mg/mouse) on d4, d7, and d10. The mice administered with only DSF and only PEG-MS-Cu nanospheres and without any treatment were used as controls.

Tumor tissues from each group were collected and fixed with 10% neutral buffered formalin solution (FUJIFILM Wako Pure Chemical Corporation, Minato City, Japan), embedded in paraffin, stained with hematoxylin and eosin (HE), and subjected to TdT-mediated dUTP nick-end labeling (TUNEL) assay at the endpoint. For the in vivo safety study, the heart, kidney, liver, lung, and spleen of mice were collected and fixed with 10% neutral buffered formalin solution, embedded in paraffin, and stained with HE at the endpoint. For the hemolysis test, the mouse red blood cells were incubated with PEG-MS-Cu nanospheres at 37 °C in saline for 1 h. The supernatant was collected after centrifugation at 3200 rpm for 5 min, and the absorbance of hemoglobin was measured at 415 nm using a microplate reader.

### 2.8. Statistical Analysis

Statistical analysis was performed using ANOVA with Tukey’s multiple comparisons post hoc test. A *p* value of <0.05 was considered statistically significant.

## 3. Results and Discussion

### 3.1. Physicochemical Characterization of MS-Cu Nanospheres

**Figure 1:**
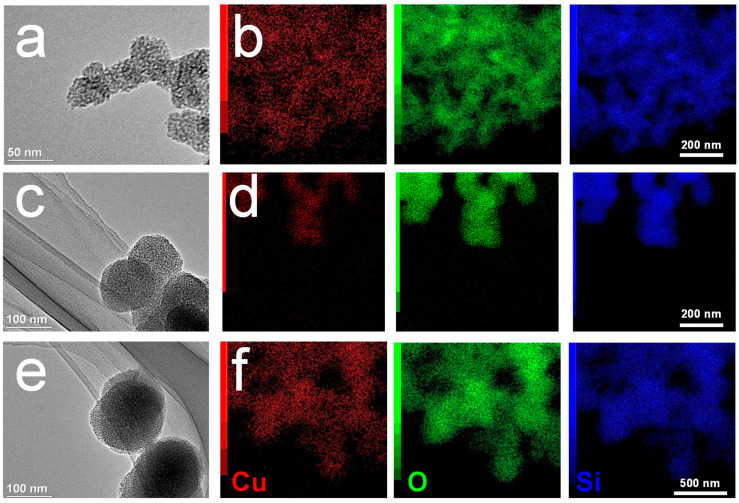
TEM (**a**,**c**,**e**) and STEM-EDX (**b**,**d**,**f**) images of MS-Cu nanospheres with different particle size. MS-Cu-1 (**a**,**b**), MS-Cu-2 (**c**,**d**), MS-Cu-3 (**e**,**f**).

**Figure 2:**
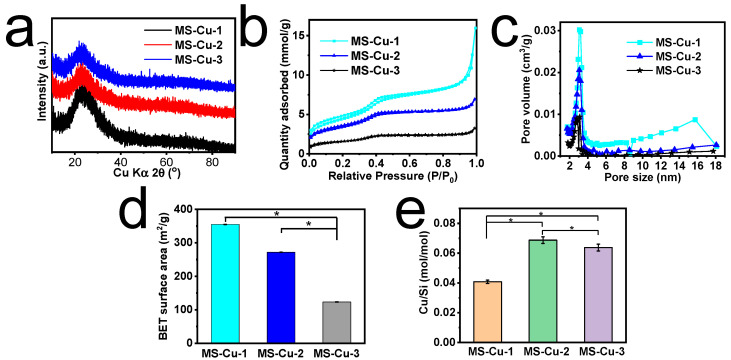
Physicochemical characterization of MS-Cu nanospheres with different particle size. XRD patterns (**a**), N_2_ adsorption-desorption isotherms (**b**), pore size distributions (**c**), BET surface areas (**d**), and Cu/Si mol ratio (**e**) of MS-Cu-1, MS-Cu-2, and MS-Cu-3 (*, *p* < 0.05).

MS-Cu nanospheres were synthesized using TEOS, CTAT, TEA, and Cu(NO_3_)_2_·3H_2_O by a one-pot method. The MS-Cu-1, MS-Cu-2, and MS-Cu-3 nanospheres showed diameters of 30–40 nm, 60–80 nm, and 100–150 nm, respectively (Figure 1). The component elements of the nanospheres were mostly Si and O, and a small amount of Cu; their Cu/Si molar ratio was 0.041–0.069 (Figure 2e). Cu was uniformly detected together with Si and O in their STEM-EDX images (Figure 1). A broad peak around 20–30° in the X-ray diffraction (XRD) patterns indicated that these nanospheres were mainly composed of amorphous silica (Figure 2a). The nanospheres showed mesopores of 2–4 nm and a BET surface area of 123–355 m^2^/g (Figure 2b–d). All these results suggest that Cu was uniformly immobilized in the amorphous MS nanospheres in MS-Cu-1, MS-Cu-2, and MS-Cu-3. In an acetate buffer, MS-Cu nanospheres exhibited a sustained release of Cu ions with an initial release rate of 13.8–16.1 μg/mL at 1 h, followed by a cumulative release rate of up to approximately 43.8–54.6 μg/mL within 2 days (Figure S1).

### 3.2. In Vitro Cytotoxicity of MS-Cu Nanospheres and DSF

**Figure 3:**
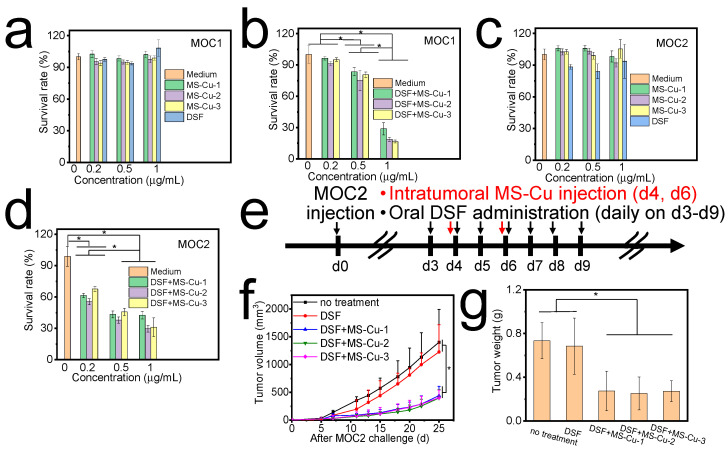
Cytotoxicity of only MS-Cu nanospheres (**a**,**c**), only DSF (**a**,**c**), and combination of MS-Cu nanospheres and DSF (**b**,**d**)against MOC1 (**a**,**b**) and MOC2 (**c**,**d**) cells in vitro. In vivo antitumor efficacy of combined oral administration of DSF and intratumoral administration of MS-Cu nanospheres. Experimental protocol (**e**), tumor volume (**f**), and tumor weight at the endpoint (**g**) (*, *p* < 0.05).

The combination of MS-Cu nanospheres and DSF significantly inhibited MOC1 and MOC2 cell growths even at MS-Cu nanosphere and DSF concentrations as low as 0.2, 0.5, and 1 μg/mL (Figure 3b,d). In particular, DSF in combination with MS-Cu-1, MS-Cu-2, and MS-Cu-3 nanospheres at 1 μg/mL limited MOC1 survival rates to 28.9 ± 5.8%, 18.6 ± 1.8%, and 16.6 ± 1.4% and MOC2 survival rates to 42.6 ± 3.5%, 29.5 ± 3.2%, and 31.1 ± 8.9%, respectively (Figure 3b,d). In contrast, only MS-Cu nanospheres and only DSF showed almost no cytotoxic efficacy at the same concentration level (Figure 3a,c). MS-Cu and PEG-MS-Cu nanospheres with concentrations of 1–10 μg/mL did not show obvious cytotoxic efficacy against NIH3T3 cells (Figure S2). MS-Cu and PEG-MS-Cu nanospheres with concentrations of 1–10 μg/mL slightly increased the ROS level compared with those without nanospheres (Figure S3).

Herein, only DSF and only MS-Cu nanospheres showed little cytotoxicity, whereas the combination of MS-Cu nanospheres and DSF showed high cytotoxic efficacy against MOC1 and MOC2 cells at concentrations of 0.2–1 μg/mL. The present results were in accordance with previous reports indicating that CuETs significantly inhibited tumor growth [15].

### 3.3. In Vivo Antitumor Efficacy of Combined Oral Administration of DSF and Intratumoral Administration of MS-Cu Nanospheres

In vivo antitumor efficacy was first studied with the combined oral administration of DSF and the intratumoral administration of MS-Cu nanospheres. Mice administered with only DSF and without any treatment showed rapid MOC2 tumor growth with tumor volumes reaching over 1000 mm^3^ and weights reaching over 0.6 g on d25 (Figure 3f,g). There was no obvious difference in tumor growth between mice administered with only DSF and those without any treatment. In contrast, the combined oral administration of DSF and intratumoral administration of MS-Cu nanospheres considerably delayed the tumor growth; tumor volumes and weights were still less than 500 mm^3^ and 0.3 g on d25. There was no obvious difference in tumor growth among those treated with MS-Cu-1, MS-Cu-2, and MS-Cu-3 nanospheres.

### 3.4. In Vivo Antitumor Efficacy and Safety of Combined Oral Administration of DSF and Intravenous Administration of PEG-MS-Cu Nanospheres

**Figure 4:**
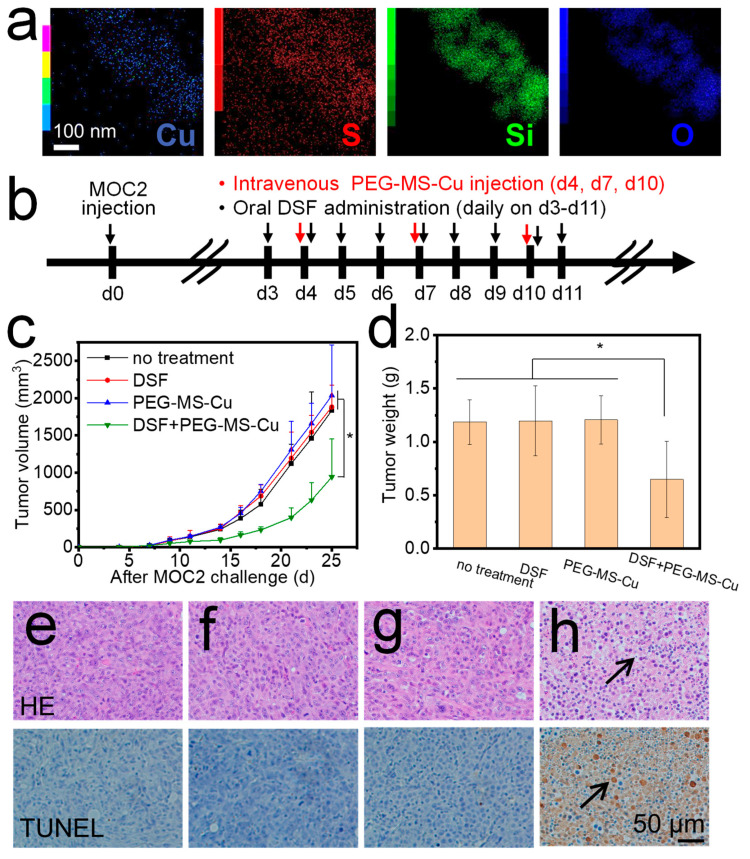
Combination of oral administration of DSF and intravenous administration of PEG-MS-Cu nanospheres inhibited MOC2 cell growth in vivo. STEM-EDX images of PEG-MS-Cu nanospheres (**a**). In vivo antitumor efficacy of combined oral administration of DSF and intravenous administration of PEG-MS-Cu nanospheres. Experimental protocol (**b**), tumor volume (**c**), and tumor weight at the endpoint (**d**). HE and TUNEL staining of tumor with no treatment (**e**), after only oral administration of DSF (**f**), only intravenous administration of PEG-MS-Cu nanospheres (**g**), and combined oral administration of DSF and intravenous administration of PEG-MS-Cu nanospheres (**h**) (*, *p* < 0.05).

For intravenous administration, the MS-Cu-1 nanospheres were further modified with PEG acid disulfide. The resulting nanospheres (PEG-MS-Cu) contained S, a component element of PEG acid disulfide, in addition to Cu, Si, and O (Figure 4a), suggesting the presence of PEG coating on their surfaces. The PEG-MS-Cu were well dispersed in ultrapure water with good stability (Figure S4).

Mice without any treatment, with the only oral administration of DSF and with the only intravenous administration of PEG-MS-Cu nanospheres, showed rapid MOC2 growth; tumor volumes reached over 1800 mm^3^ and weight reached over 1.1 g on d25 (Figure 4c,d). These results indicate that either oral administration of DSF or intravenous administration of PEG-MS-Cu nanospheres did not have a significant cytotoxic effect against tumor cells. However, the combined oral administration of DSF and intravenous administration of PEG-MS-Cu nanospheres considerably delayed the tumor growth speed; tumor volumes and weights were still less than 1000 mm^3^ and 0.7 g on d25 (Figure 4c,d). This group showed a significantly lower weight of tumor at the endpoint than the other three groups (Figure 4d). The combined oral administration of DSF and intravenous administration of PEG-MS-Cu nanospheres (Figure 4c,d) showed relatively weaker antitumor efficacy than the combined oral administration of DSF and intratumoral administration of MS-Cu nanospheres (Figure 3f,g), although the experimental parameters were not the same. This was due to a limited amount of intravenously administered PEG-MS-Cu nanospheres reached the tumor site (Figure S5).

To maximize the chemotherapeutic efficacy of DSF, it is highly desirable to deliver a sufficient amount of Cu^2+^ ions selectively to the tumor site [15,21,22,34,35,36]. However, oral Cu^2+^ administration may cause a low therapeutic efficacy and an undesirable toxicity originating from insufficient Cu^2+^ accumulation at the tumor site and nonspecific Cu^2+^ accumulation in normal tissues [23,24]. In a previous study, Cu^2+^ and DSF containing nanoparticles were used as Cu^2+^ and DSF supply systems to tumors. Cu^2+^ and DSF were rapidly released in an acid tumor microenvironment after endocytosis and degradation, which caused CuETs and ROS generation within the tumor. As a result, the Cu^2+^ and DSF containing nanoparticles showed high chemotherapeutic efficacy against tumors [22]. It is preferable to deliver Cu compounds to the tumor site by using nanoparticles and to deliver DSF by means of clinically approved oral administration. We herein construct MS-Cu nanospheres as the exogenous copper supply system for the delivery of Cu^2+^ ions into the tumor microenvironment. The nanospheres delivered to the tumor site can release Cu^2+^ ions locally in the tumor microenvironment [22,26]. In combination with the clinically approved oral administration of DSF, CuETs can be synthesized in the tumor microenvironment, thus showing a cytotoxic effect against tumors [14,36,37].

Although considerable progress had been made on targeted therapy, it has been reported that only several percent of systemically administered chemotherapy drugs can reach the tumor site, generally resulting in serious side effects and even toxicity to normal tissues, as well as the limited use of chemotherapy drugs and unsatisfactory treatment outcomes [38,39]. With the improvement of technology in material science, image-guided biopsies, and injections, intratumoral administration is now a feasible, safe, and increasingly popular clinical approach for cancer [40,41]. Intratumoral administration has shown considerable advances over systemic administration, since it provides a safer and more efficient, durable, and aggressive administration of chemotherapy drugs directly into the tumor site [42,43]. Considering the limited diffusion distance of chemotherapy drugs in tumors, generally, one to two intratumoral administrations are required for tumors smaller than 4 cm^3^, whereas as many intratumoral administrations as possible are required for larger tumors, considering patient tolerance and tumor accessibility [44]. Multiple intratumoral administrations are still likely to be accompanied by an increased leakage risk of chemotherapy drugs to surrounding normal tissues, thus causing undesired side effects and toxicity to normal tissues [45].

Herein, oral DSF administration was combined with MS-Cu intratumoral or PEG-MS-Cu intravenous administration, which both showed antitumor efficacy against MOC2 cells in vivo (Figure 3f,g and Figure 4c,d). DSF is a disulfide dimer that can be metabolized to dithiocarbamate (DTC) in a physiological environment. DTC contains reactive thiol nucleophiles and is an efficient chelator for various ions. In particular, chelating Cu^2+^ with DTC results in the synthesis of CuETs, which show a markedly improved antitumor efficacy compared with the original DSF [14,36,37]. In particular, the combination of oral DSF administration and MS-Cu intratumoral administration is promising for reducing the chemotherapy-related toxicity based on two mechanisms. First, the combination of oral DSF administration and MS-Cu intratumoral administration maximizes the MS-Cu concentrations in tumor sites, while it minimizes non-target MS-Cu exposure to normal tissues. Second, even a small amount of MS-Cu may be released to surrounding normal tissues. The release of Cu^2+^ ions under a normal tissue pH of approximately 7.4 might be limited [22,26], and as a result the synthesis of CuETs by Cu^2+^ ions and DSF in normal tissues would be markedly inhibited.

To further confirm the cytotoxic efficacy, tumor tissues from each group were collected at the endpoint, fixed, and stained with HE and TUNEL. The combination of the oral administration of DSF and the intravenous administration of PEG-MS-Cu nanospheres caused an obvious tumor cell apoptosis with apparent nuclei shrinkage and fragmentation (Figure 4h, indicated by black arrows). For mice without any treatment, with the only oral administration of DSF and with the only intravenous administration of PEG-MS-Cu nanospheres, no obvious tumor apoptosis was observed (Figure 4e–g). The HE and TUNEL stain results were in accordance with the tumor growth curve and tumor weight results shown in Figure 4c,d.

**Figure 5:**
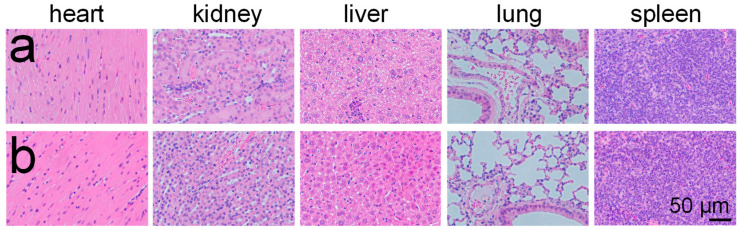
Combination of oral administration of DSF and intravenous administration of PEG-MS-Cu nanospheres showed no obvious toxicity to normal tissues in vivo. Histological sections of heart, kidney, liver, lung, and spleen of mice without any treatment (**a**), and with combined oral administration of DSF and intravenous administration of PEG-MS-Cu nanospheres (**b**).

The PEG-MS-Cu nanospheres did not show hemolysis at concentrations of 0–10 μg/mL in vitro (Figure S6). For in vivo safety profiles, the heart, kidney, liver, lung, and spleen were collected at the endpoint from mice without any treatment and with the combination of the oral administration of DSF and the intravenous administration of PEG-MS-Cu nanospheres. No significant damage was observed in any tested tissues (Figure 5), suggesting that the present combined medication is unlikely to cause serious side effects on these normal tissues [22,25].

## 4. Conclusions

To fulfil the therapeutic potential of DSF-based chemotherapy in cancer treatment, MS-Cu nanospheres were developed as the exogenous copper supply system for the efficient delivery of Cu^2+^ ions into the tumor microenvironment. The synthesized MS-Cu nanospheres showed diameters of 30–40 nm, 60–80 nm, and 100–150 nm, and were composed of Si, O, and Cu with Cu/Si molar ratios of 0.041–0.069. Cu was uniformly detected together with Si and O in their STEM-EDX images. Only DSF and only MS-Cu nanospheres showed little cytotoxicity in vitro, whereas the combination of DSF and MS-Cu nanospheres showed MOC1 survival rates of 16.6–28.9% and MOC2 survival rates of 29.5–42.6% at concentrations of 1 μg/mL. Oral DSF administration in combination with MS-Cu nanospheres intratumoral or PEG-MS-Cu nanospheres intravenous administration showed significant antitumor efficacy against MOC2 cells in vivo. The combination of the oral administration of DSF and the intravenous administration of PEG-MS-Cu nanospheres caused an obvious tumor cell apoptosis with apparent nuclei shrinkage and fragmentation as shown by the HE and TUNEL stain results. In this study, we demonstrated a strategy for the in situ transformation of low-toxicity/nontoxic DSF into a toxic chemotherapy drug, CuET, in the tumor microenvironment with the aid of exogenous copper, thus succeeding in maximizing the strategy’s therapeutic efficacy while minimizing the side effects. Further studies on Cu metabolism in the body, the efficiency and yield of in situ CuET synthesis, long-term safety, efficacy in other tumors, and so forth are required for clinical application.

## Data Availability

The data presented in this study are available on request from the corresponding author.

## Supplementary Materials

The following supporting information can be downloaded at: https://www.mdpi.com/article/10.3390/pharmaceutics15041316/s1, Figure S1: In vitro copper ion release from MS-Cu nanospheres; Figure S2: In vitro safety of MS-Cu and PEG-MS-Cu nanospheres; Figure S3: In vitro reactive oxygen species generation by MS-Cu and PEG-MS-Cu nanospheres; Figure S4: The particle size of PEG-MS-Cu nanospheres tested by dynamic light scattering analysis immediately (left) and 3d (right) after ultrasonication; Figure S5: In vivo distribution of PEG-MS-Cu nanospheres; Figure S6: Hemolysis of PEG-MS-Cu nanospheres.
