# Supplementary material for: In Situ Synthesis of a Tumor-Microenvironment-Responsive Chemotherapy Drug
Source: Pharmaceutics. 2023 Apr 21;15(4):1316. doi: 10.3390/pharmaceutics15041316 (PMC10141230; doi:10.3390/pharmaceutics15041316)
Supplement: Supplementary file 1 [file pharmaceutics-15-01316-s001.zip › pharmaceutics-2316490-supplementary.pdf]

pharmaceutics

MDPI

# Supplementary Material: In situ synthesis of tumor-microenvironment-responsive chemotherapy drug

**Xiupeng Wang, Ayako Oyane, Tomoya Inose, Maki Nakamura**

## S1. Supplementary data

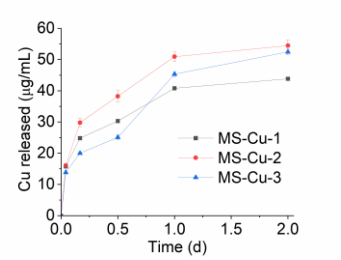


**Figure S1**. In vitro copper ion release from MS-Cu nanospheres

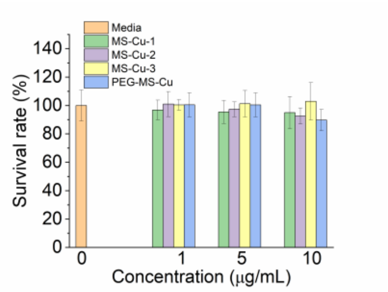


**Figure S2**. In vitro safety of MS-Cu and PEG-MS-Cu nanospheres

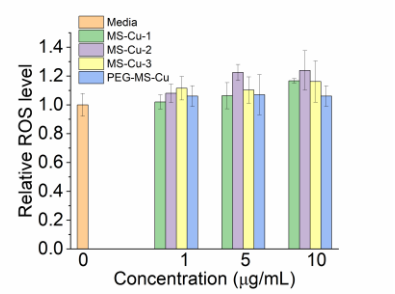


**Figure S3**. In vitro reactive oxygen species generation by MS-Cu and PEG-MS-Cu nanospheres

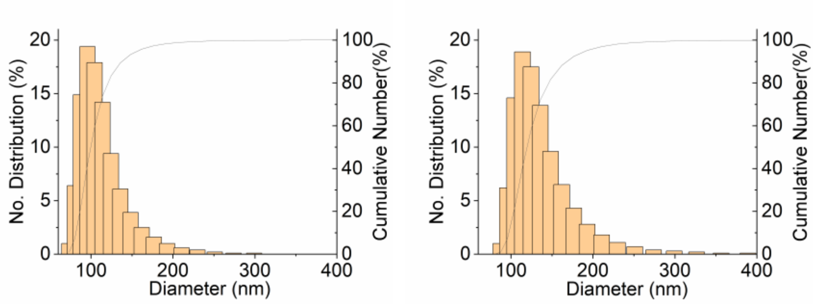


**Figure S4.** The particle size of PEG-MS-Cu nanospheres tested by dynamic light scattering analysis immediately (left) and 3d (right) after ultrasonication.

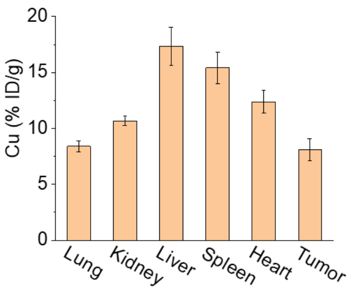


**Figure S5**. In vivo distribution of PEG-MS-Cu nanospheres

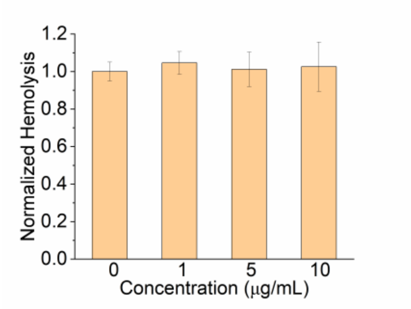


**Figure S6**. Hemolysis of PEG-MS-Cu nanospheres
